# Petholeum Extract of Bile for Treatment of Tuberculous Lesions

**Published:** 1914-08

**Authors:** 


**Petholeum Extract of Bile for Treatment of Tuberculous Lesions.** G. Lemoine of Paris and E. Gerard of Lille, Gaz. des Prat., May 15, 1914, advocates this treatment both to compensate for the waste of cholesterin and to aid in cicatrization. The expectoration may contain 10% of cholesterin and the waste has been found to reach as high as a quarter of a gram a day.

